# *In vitro* and *in vivo* production and split-intein mediated ligation (SIML) of circular bacteriocins

**DOI:** 10.3389/fmicb.2022.1052686

**Published:** 2022-11-14

**Authors:** Nuria Peña, Michael J. Bland, Ester Sevillano, Estefanía Muñoz-Atienza, Irene Lafuente, Mohamed El Bakkoury, Luis M. Cintas, Pablo E. Hernández, Philippe Gabant, Juan Borrero

**Affiliations:** ^1^Sección Departamental de Nutrición y Ciencia de los Alimentos, Facultad de Veterinaria, Universidad Complutense de Madrid (UCM), Madrid, Spain; ^2^Syngulon SA, Seraing, Belgium

**Keywords:** circular bacteriocin, split-intein, *in vitro*-cell free protein synthesis, synthetic biology, circularization, antimicrobial peptide

## Abstract

Circular bacteriocins are antimicrobial peptides produced by bacteria that after synthesis undergo a head-to-tail circularization. Compared to their linear counterparts, circular bacteriocins are, in general, very stable to temperature and pH changes and more resistant to proteolytic enzymes, being considered as one of the most promising groups of antimicrobial peptides for their potential biotechnological applications. Up to now, only a reduced number of circular bacteriocins have been identified and fully characterized, although many operons potentially coding for new circular bacteriocins have been recently found in the genomes of different bacterial species. The production of these peptides is very complex and depends on the expression of different genes involved in their synthesis, circularization, and secretion. This complexity has greatly limited the identification and characterization of these bacteriocins, as well as their production in heterologous microbial hosts. In this work, we have evaluated a synthetic biology approach for the *in vitro* and *in vivo* production combined with a split-intein mediated ligation (SIML) of the circular bacteriocin garvicin ML (GarML). The expression of one single gene is enough to produce a protein that after intein splicing, circularizes in an active peptide with the exact molecular mass and amino acid sequence as native GarML. *In vitro* production coupled with SIML has been validated with other, well described and not yet characterized, circular bacteriocins. The results obtained suggest that this synthetic biology tool holds great potential for production, engineering, improving and testing the antimicrobial activity of circular bacteriocins.

## Introduction

Bacteriocins are ribosomally synthesized antimicrobial peptides produced by bacteria, with potential applications traditionally focused on food preservation, mainly due to the widespread identification, characterization and evaluation of the antimicrobial activity of bacteriocins from lactic acid bacteria (LAB), and the approval of nisin as a food preservative by regulatory agencies (Younes et al., 2017; Soltani et al., 2021). However, during the last few years, there has been a switch in the tendencies about the envisaged biotechnological applications of bacteriocins, with an increasing number of patents and studies proposing their use as antimicrobial agents in human and animal health and other non-food industrial applications (Chikindas et al., 2018; Zimina et al., 2020; Soltani et al., 2021).

Most bacteriocins are synthesized as biologically inactive precursors or prepeptides, containing an N-terminal extension that is cleaved off during export to generate their biologically active or mature form. The mature bacteriocin peptides were initially classified into two main classes: class I or lantibiotics, with lanthionine-containing post-translationally modified amino acid residues, and class II bacteriocins with unmodified amino acid residues (Cotter et al., 2005). However, currently the class I group of modified bacteriocins includes all ribosomally synthesized and post-translationally modified peptides (RiPPS) that undergo enzymatic modification during biosynthesis, providing molecules with uncommon amino acids or structures with an impact on their properties (e.g., lanthipeptides, thiopeptides, lasso peptides, cyclized peptides, sactipeptides and others), whereas the class II group of bacteriocins, groups bacteriocins that do not require posttranslational modification enzymes for their maturation (Alvarez-Sieiro et al., 2016; Chikindas et al., 2018; Montalbán-López et al., 2021; Ongpipattanakul et al., 2022).

The head-to-tail class I cyclized bacteriocins are part of a growing family of ribosomally synthesized peptides found in mammals, plants, fungi and bacteria characterized by their N-to C-terminal covalent link forming a circular backbone (Alvarez-Sieiro et al., 2016). Circular bacteriocins are synthesized as linear precursor peptides, containing a leader peptide (2 to 35 amino acid residues long) which is cleaved off during the maturation process. The linear peptides (58 to 70 amino acid residues long) are cyclized by the formation of an amide bond between the N-and C-terminal residues, before being exported out of the cell. The majority of circular bacteriocins fall under subclass I, which possess a high isoelectric point (pI >9). Subclass II circular bacteriocins tend to feature lower isoelectric points (pI <7; (Gabrielsen et al., 2014). Characterization of the three-dimensional (3D) structures of several circular bacteriocins have revealed that they share a compact globular structure consisting of repeated α-helical motifs surrounding a hydrophobic core (Borrero et al., 2011; Van Belkum et al., 2011; Gabrielsen et al., 2014). This highly stable circular structure makes the circular bacteriocins particularly resilient, with characteristic traits such as high thermo-, pH-, and proteolytic stability. These traits make the circular bacteriocins highly promising antimicrobial peptide candidates for many human, animal and industrial applications (Towle and Vederas, 2017; Borrero et al., 2018; Perez et al., 2018).

The synthesis and secretion of circular bacteriocins involves the action of proteins encoded by genes that are usually clustered together. The organization of genes in clusters directing the production of head-to-tail cyclized bacteriocins is well conserved and includes, a minimum of 5 to 7 genes encoding the bacteriocin precursor peptide, immunity proteins, an ATP-binding protein, a protein containing a domain of unknown function 95 (DUF95) presumably involved as an immunity-associated transporter and a secretion-aiding agent, and one or more proteins of unknown function, although the detailed mechanism by which these bacteriocins circularize has yet to be determined (Mu et al., 2014; Perez et al., 2018; Major et al., 2021). Nevertheless, most studies suggest that leader peptide removal and cyclization are two separate processes (Gabrielsen et al., 2014; Perez et al., 2017). Up to now there are about 21 experimentally confirmed circular bacteriocins. However, advances in the sequencing and bioinformatic analysis of many bacterial genomes have exponentially accelerated the identification of putative novel circular bacteriocins. Accordingly, several recently published research articles have mined 1,000 of published microbial genomes for the presence of hypothetical circular bacteriocin clusters, and found a significant number of Gram positive strains carrying potential novel circular bacteriocins (Vezina et al., 2020; Xin et al., 2020; Major et al., 2021).

Traditionally, new bacteriocins have been identified after their purification in supernatants of the native strain or by their production in heterologous hosts, encoding the genes for synthesis and processing of the mature bacteriocin (Borrero et al., 2018). This process is laborious, expensive and time consuming and, in most cases, demands the presence of the native bacteriocin-producing bacteria. In 2019 we published a study describing the *in vitro* synthetic production of a collection of 164 different class II bacteriocins (PARAGEN 1.0 library), using a cell-free protein synthesis (CFPS) approach (Gabant and Borrero, 2019). While this method has proven efficient for production of the unmodified class II bacteriocins, *in vitro* production of class I circular bacteriocins is still a difficult task as they rely on the expression of proteins involved in their synthesis, processing and modification.

Split inteins have been traditionally used as synthetic biology tools for the circularization of peptides and proteins (Scott et al., 1999). In spite of this, they have not yet been applied in the bacteriocin research world. In this work, we have developed a fast and reliable method for production of active circular bacteriocins by combining an *in vitro* cell-free protein synthesis (IV-CFPS) protocol with the split-intein mediated method for ligation of peptides and proteins (SICLOPPS) method (Tavassoli and Benkovic, 2007; Townend and Tavassoli, 2016). Fusion of the C-and N-terminal intein fragments from the *Nostoc punctiforme* Npu DnaE split intein to mature garvicin ML (GarML), produced by *Lactococcus garvieae* DCC43 (Borrero et al., 2011), has allowed the synthesis and circularization of this bacteriocin without the assistance of any other gene encoding a peptide or protein. The production and circularization of GarML with antimicrobial activity has been determined both with an IV-CFPS system and *in vivo* in *Escherichia coli* cells. The evaluation, by MALDI-TOF MS and targeted proteomics combined with massive peptide analysis of the peptide produced, confirmed the production and circularization of an active antimicrobial GarML with the same aminoacid sequence and molecular mass of the native bacteriocin, produced by *L. garvieae* DCC43. This synthetic biology approach has been also successfully applied for the IV-CFPS and split-intein mediated ligation (SIML) of 26 more, well described and not yet characterized, circular bacteriocins.

## Materials and methods

### Bacterial strains, gene constructions, and culture conditions

**Figure 1:**
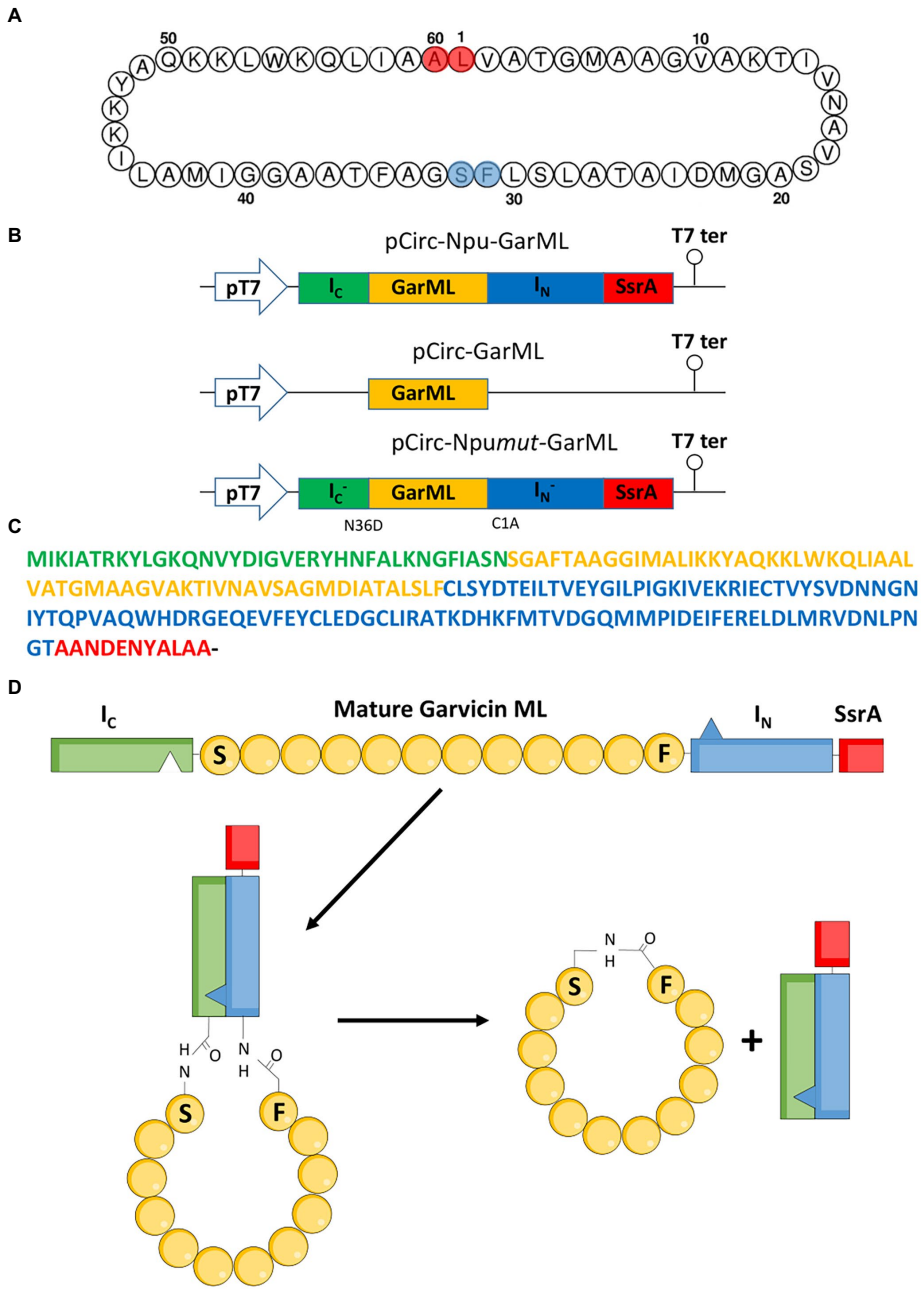
Garvicin ML circularization by the split-intein mediated ligation (SMIL) of peptides and proteins. **(A)** Amino acid sequence of mature GarML; the amino acids implicated in the head-to-tail circularization of native GarML, in red, and the amino acids selected for the IV-CFPS and SIML and the *in vivo* production and circularization of GarML, in blue. **(B)** Schematic representation of the pUC-derived protein expression vectors encoding the Npu-GarML, GarML and Npu*mut*-GarML gene products. **(C)** Amino acid sequence of the construct I_c_-GarML-I_N_-SsrA encoded by pCirc-Npu-GarML; the C-terminal-36-amino acid sequence (I_c_) and the N-terminal-104-amino acid sequence (I_N_) of the Npu split intein are shown in green and blue, respectively; the GarML sequence is in yellow and the SsrA sequence in red. **(D)** Schematic representation of the cyclization process of GarML; the intein peptides, I_N_ and I_c_, interact to form an active peptide that splices including circularization of mature GarML.

The bacterial strains and plasmid constructions used in this work, are listed in Table 1. *E. coli* DH5α (Thermo Fisher Scientific, WI, United States) and *E. coli* BL21 (DE3) (Invitrogen S.A., Barcelona, Spain) were grown in Luria Bertani broth (Sigma-Aldrich Inc., St. Louis, MO, United States) at 37°C with ampicillin at 100 μg/mL and shaking (250 rpm)*. Lactococcus garvieae* DCC43, *L. garvieae* CECT5806 and *Lactococcus lactis* IL1403 were grown in MRS broth (OXOID [Thermo Fisher Scientific]) at 32°C. The description and amino acid sequence of the components used for the SIML of the bacteriocins used in this study, are shown in Table 2. An overview of the strategy employed for ligation and circularization of GarML by the split-intein mediated ligation of peptides and proteins (SICLOPPS) system, is shown in Figure 1. The producer strains and mature amino acid sequences of the bacteriocins evaluated for their IV-CFPS and SIML, are shown in the Supplementary material. A synthetic gene construct containing the C-and N-terminal intein fragments from the *Nostoc puntiforme* Npu DnaE split intein (I_C_ and I_N_, respectively), flanking the gene coding the mature peptide of GarML was synthesized by Genewiz (Agenta Life Technologies, South Plainsfield, NJ, United States). Serine (S32) was the first amino acid residue in the linear conformation (S1 in the new conformation) and phenylalanine (F31) as the last residue (F60 in the new conformation) (Figure 1A). In addition a protein degradation tag (SsrA) was included in the C-terminus of the gene construct (Figures 1B–D; Table 2). Once the complete linear novel amino acid sequence of GarML was designed, it was reverse-translated according to the codon usage of *E. coli (*www.bioinformatics.org/sms2/rev_trans.html*)*, and placed under the control of a pUC-derived protein expression vector (pCirc-Npu-GarML) containing the T7 promoter region, a start codon (ATG), a stop codon (TAA) and a T7 terminator region. In addition, two control protein expression constructs were synthesized: one for production of the S1-F60 linear GarML without inteins (pCirc-GarML), and another for the production of the linear S1-F60 GarML flanked by I_C_ and I_N_ with one amino acid substitution each, N36D and C1A, respectively (pCirc-Npu*mut*-GarML) (Figure 1B; Table 2). The designed and constructed pCirc-derived vectors for the IV-CFPS and SIML of circular bacteriocins, are shown in the Supplementary material. Gene synthesis was performed by Genewiz.

**Table 1:** Strains and plasmids used in this study.

**Strain or plasmid**	**Description**	**Source and/or References**^**a**^
**Strains**		
*Escherichia coli* DH5α	Stabilization of recombinant plasmids	Thermo Fisher Scientific
*Escherichia coli* BL21 (DE3)	Production of Npu-GarML	Thermo Fisher Scientific
*Lactococcus garvieae* DCC43	Garvicin ML producer	Borrero et al. (2011)
*Lactococcus garvieae* CECT5806	Indicator sensitive to GarML	CECT
*Lactococcus lactis* IL1403	Indicator sensitive to different bacteriocins	Chopin et al. (1984)
**Plasmids**		
pCirc-Npu-GarML	Amp^r^; T7 plasmid carrying I_C_ – *garML* – I_N_ – SsrA	Genewiz
pCirc-GarML	Amp^r^; T7 plasmid carrying *garML*	Genewiz
pCirc-Npu*mut*-GarML	Amp^r^; T7 plasmid carrying I_C_^−^ – *garML* – I_N_^−^- SsrA	Genewiz

a CECT, Colección Española de Cultivos Tipo, Valencia (Spain).

**Table 2:** Components of the split-intein mediated ligation (SIML) of circular bacteriocins, used in this study.

**Fragment**	**Description**	**Amino acid sequence**	**References**
**I**_C_	C-terminal intein fragment from *Nostoc punctifrome* (*Npu*) DnaE split intein	MIKIATRKYLGKQNVYDIGVERYHNFALKNGFIASN	Iwai et al. (2006)
**I**_N_	N-terminal intein fragment from *Nostoc punctifrome* (*Npu*) DnaE split intein	CLSYDTEILTVEYGILPIGKIVEKRIECTVYSVDNNGNIYTQPVAQWHDRGEQEVFEYCLEDGCLIRATKDHKFMTVDGQMMPIDEIFERELDLMRVDNLPNGT	Iwai et al. (2006)
**I**_C_^−^	C-terminal intein fragment from *Nostoc punctifrome* (*Npu*) DnaE split intein with a point substitution of essential residue N36 by a D	MIKIATRKYLGKQNVYDIGVERYHNFALKNGFIAS**D**	Cheriyan et al. (2013)
**I**_N_^−^	N-terminal intein fragment from *Nostoc punctifrome* (*Npu*) DnaE split intein with a point substitution of essential residue C1 by an A	**A**LSYDTEILTVEYGILPIGKIVEKRIECTVYSVDNNGNIYTQPVAQWHDRGEQEVFEYCLEDGCLIRATKDHKFMTVDGQMMPIDEIFERELDLMRVDNLPNGT	Cheriyan et al. (2013)
**SsrA**	Protein degradation tag	AANDENYALAA	Keiler et al. (1996)

In bold the amino acid residue substitutions generating non-functional inteins are depicted.

### *In vitro* cell-free protein synthesis and split-intein mediated ligation of circular bacteriocins, and determination of their antimicrobial activity

All the pCirc-derived recombinant vectors, including pCirc-Npu-GarML, pCirc-GarML, pCirc-Npu*mut*-GarML and the pCirc-derived vectors shown in the Supplementary material, and encoding the intein peptides fused to the appropriate mature bacteriocins, were stabilized by their transformation into competent *E. coli* DH5α cells. The amplified plasmids were purified from *E. coli* DH5α by the NuceloSpin Plasmid kit (Macherey-Nagel, Düren, Germany). They were further used as templates for the IV-CFPS of the resulting peptides by using the PURExpress® *in vitro* Protein Synthesis Kit (New England Biolabs, Massachusetts, United States), as previously described (Gabant and Borrero, 2019). The antimicrobial activity of the samples was evaluated by the spot-on-agar test (SOAT), with 5 μl of the IV-CFPS-produced samples deposited on the surface of petri plates, previously inoculated with the bacterial indicator strain *L. lactis* IL1403 (Gabant and Borrero, 2019). Samples showing a clear halo of inhibition were considered as containing active bacteriocins.

### *In vivo* production and purification of garvicin ML from *Escherichia coli* BL21 (DE3) (pCir-Npu-GarML)

For production and purification of the antimicrobial activity of competent *E. coli* BL21 (DE3), transformed with pCirc-Npu-GarML, an initial inoculum of the recombinant *E. coli* BL21 (DE3) (pCirc-Npu-GarML) cells was added in 10 mL of LB broth with ampicillin at 100 μg/ml (LB-Amp), and grown overnight at 37°C in a shaking incubator. Later, 500 mL of fresh LB-Amp were inoculated with the overnight grown *E. coli* recombinant cells, until an OD_600_ attained of 0.1, in a shaking incubator at 37°C. When the grown cells reached an OD_600_ of, approximately 0.4, the isopropyl-β-D-thiogalactoside (IPTG) inducer was added to a final concentration of 0.5 mM. The culture was grown for another 3 h, and the cells pelleted by centrifugation at 8,000 g for 15 min at 4°C. The cells were resuspended in 20 mL ice-cold 20 mM phosphate buffer, pH 6.0 with 1 M NaCl, and lysed by sonication (6 cycles of 10 s at 45% maximum intensity, with 1 min incubation in ice in between the cycles) in a 450 Digital Sonifier (Branson Ultrasonics, Connecticut, United States). The insoluble cell debris was pelleted by centrifugation at 8,000 g, for 15 min at 4°C, and the resulting cellular soluble fraction (CSF) filtered through a 0.45 μm filter (Sartorius, Goettingen, Germany).

Ammonium sulfate (10% w/v) was added to the CSF which was further deposited on a column with 2 mL of Octyl Sepharose CL-4B (GE Healthcare, Chicago, United States), previously washed with dH_2_O and equilibrated with 15 mL of 20 mM phosphate buffer, pH 6.0 with ammonium sulfate (1% w/v), named equilibration buffer (EB). The SF was added to the column and washed with 10 mL EB. The antimicrobial activity was eluted from the column with 10 mL 70% ethanol diluted in 20 mM phosphate buffer, pH 6.0. Finally, the fraction eluted from the hydrophobic interaction support was diluted 5 times in dH_2_O + trifluoroacetic acid (TFA, 0,1% v/v), and subjected to reversed-phase chromatography in a Source 5 RPC ST4.6–150 column (GE Healthcare, Chicago, United States) in a Fast-Protein Liquid Chromatography System (ÄKTA, RP-FPLC). Samples were eluted with a mobile phase of TFA 0.1% (v/v) in a mixture of water (eluent A) and isopropanol (eluent B). The compounds in the column were initially eluted with 100% eluent A for 5 min, then with a linear gradient of 0–70% eluent B over 50 min, followed by a linear gradient to 100% eluent B over 5 min and 100% eluent B for 7 min. The flow rate was maintained at 0.5 mL/min, the absorbance monitored at 254 nm and the column kept at room temperature. All the eluted RP-FPLC fractions were assayed for antimicrobial activity by the SOAT method (Gabant and Borrero, 2019), with *L. garvieae* CECT5806 as the indicator organism. The RP-FPLC fractions displaying antimicrobial activity were subjected to a second run of RP-FPLC, under the same conditions.

### Determination of the molecular mass of the *in vitro* and *in vivo* produced recombinant garvicin ML

A total of 1 μL of each sample was spotted onto a MALDI target plate and allowed to air-dry at room temperature. Then, 0.8 μl of Sinapic acid matrix (Sigma-Aldrich Inc.) in 30% acetonitrile 0.3% trifluoroacetic acid were added and allowed to air-dry at room temperature again. MALDI-TOF analyses were performed using a 4,800 Plus Proteomics Analyzer MALDI-TOF/TOF mass spectrometer (Applied Biosystems/MDS Sciex, Toronto, Canada). MALDI-TOF mass spectra were acquired in a range of masses of 900–6,000 Da with the linear medium mass acquisition method in the positive mode using an accelerating voltage of 20 kV. Default and plate calibration were performed using the calibration mixture 2 Peptide Mass Standards Kit (Applied Biosystems/MDS Sciex).

### Identification of the *in vivo* produced garvicin ML by targeted proteomics combined with massive peptide analysis

The eluted antimicrobial fractions from the purified GarML, produced by *E. coli* BL21 (DE3) (pCirc-Npu-GarML), were evaluated for identification and characterization of the purified bacteriocin at the Unidad de Proteómica, Universidad Complutense de Madrid (UCM, Madrid, Spain). The proteins in samples were digested with trypsin in S-Trap™ micro columns (ProtiFI, NY, United States) according to manufacturer’s instructions. Briefly, proteins were reduced with 10 mM dithiothreitol (DTT) at 56°C for 60 min and then alkylated with 25 mM iodacetamide for 60 min. Sodium dodecyl sulfate (SDS) at 20% and tetraethylammonium bromide (TEAB) 1 M were then added, and the samples further acidified with phosphoric acid at 12%. The samples were then loaded into S-Trap™ micro columns (ProtiFI, NY, United States) with the S-Trap protein binding buffer (STBB) at a ratio of 6:1, and digested with 1.5 μg of recombinant trypsin (Roche Molecular Biochemicals, NJ, United States) in 50 mM TEAB at 47°C for 90 min. Finally, the resulting peptides were eluted, dried in a SpeedVac™ concentrator (Thermo Fisher Scientific), reconstituted at their initial volume with 2% acetonitrile (ACN) and 0.1% formic acid (FA), and quantified in a Qubit Fluorometer (Thermo Fisher Scientific)..

Next, approximately 1 μg of the peptides was analyzed by nano-liquid chromatography (nano Easy-nLC 1,000) coupled to a high-resolution Q-Exactive HF mass spectrometer (Thermo Fisher Scientific). Peptides were concentrated “on-line” by reversed-phase chromatography (RP) using an Acclaim PepMap 100 column (Thermo Fisher Scientific) and then separated into a C18 Picofrit column (Easy Spray Column, PepMap RSLC C18n, Thermo Fisher Scientific) with integrated spray tip, operating at a flow rate of 250 nl/min. The peptides were then eluted using a gradient from 2 to 35% buffer A (0.1% AF in dH_2_O) for 90 min and up to 45% buffer B (0.1% AF in ACN) in 10 more min. A combined targeted proteomics method including a data-dependent acquisition (DDA) plus an inclusion list with the m/z of fragments from the *in silico* digestion of the mature circular GarML was obtained to detect the peptides of interest as well as other proteins in the samples. The inclusion list were obtained with the free program Skyline v. 20.2 (https://skyline.gs.washington.edu). The peptides were detected in full scan MS mode with a resolution of 60,000 over a mass range m/z of 350–1800 Da. In each MS microscan, up to 15 precursors with a charge from +2 to +4 were selected depending on their intensity (threshold: 1.3 × 10^4^) with dynamic exclusion of 10 s, followed by isolation with a window width of +/− 2 units of m/z, during a maximum time of 120 ms, for fragmentation by HCD (High Collision Dissociation) with a normalized collision energy of 20%. The MS/MS spectra were acquired with a resolution of 30,000 in positive mode.

Peptide and protein identification from MS/MS data were carried out with Proteome discoverer 2.4 Software (Thermo Scientific) using MASCOT v 2.6 or Sequence HT search engines and Peaks Studio v 10.5 Software (Bioinformatic solution Inc.)(Ma et al., 2003). The following databases were used: SwissProt (562,755 sequences) downloaded from Uniprot (https://www.uniprot.org), annotated proteins of *Lactococcus garvieae* (4,386 sequences) downloaded from NCBI (https://www.ncbi.nlm.nih.gov/), all predicted sequences after GarML digestion and the Contaminants DB (247 sequences) including the most common contaminants, (www.matrixscience.com/help/seq_db_setup_contaminants.html). The following parameters were used for the searches: tryptic cleavage, up to two missed cleavage sites allowed, Mass tolerances of 10 ppm for precursor ions and 0.02 Da for MS/MS fragment ions and the searches were performed allowing optional Methionine oxidation and acetylation protein N terminal, and as fixed modification, carbamidomethylation of Cysteine. The false discovery rates (FDR) scores were adjusted by a percolator algorithm. The acceptances criteria for peptide/proteins identification were a FDR < 1% and at least one peptide identified with high confidence. And in Peaks Software were PSM-FDR < 1%; protein-1OlogP ≥ 20 with at less one unique confident peptide. *De novo* sequences or tags were filter by ALC score ≥ 80 and peptide modified by AScore ≥20.

## Results

### Genetic design of the peptide constructs

A synthetic gene construct containing the C-and N-terminal intein fragments of the *Nostoc punctiforme* Npu DnaE split intein (I_C_ and I_N_, respectively; Townend and Tavassoli, 2016), flanking the gene coding the mature GarML was synthesized. Native GarML circularization occurs after a head-to-tail ligation between the amino acid residues leucine (L1) and alanine (A60) and cleavage of the leader sequence (Borrero et al., 2011; Figure 1A). The mechanism of split-intein mediated circularization of peptides and proteins (SICLOPPS) splicing involves formation of the active intein through association of I_C_ and I_N_, followed by splicing to give the cyclic peptide product and the liberation of I_C_ and I_N_ as the byproducts. Similarly, the intein chemistry requires the first amino acid of the target peptide to be, preferably, either a nucleophilic cysteine (C) or a serine (S) residue (Townend and Tavassoli, 2016). Since GarML has no cysteines in its mature amino acid sequence but it has 3 serines (S19, S29 and S32), then the order of the amino acid residues in the designed novel GarML was modified, selecting S32 as the first residue in the linear novel conformation (S1 in the new conformation) and phenylalanine (F31) as the last residue (F60 in the new conformation) (Figure 1A). In addition, a protein degradation tag (SsrA) was included in the C-terminus of the gene construct to overcome the potential toxicity of the Npu intein after splicing (Figures 1B–D; Table 2) (Townend and Tavassoli, 2016). Once the novel amino acid sequence of the linear GarML was designed, it was reverse-translated according to the codon usage of *E. coli*, and placed under the control of a T7 promoter in a pUC-derived protein expression vector (pCirc-Npu-GarML). In addition, two control protein expression vectors were synthesized: one for production of the S1-F60 linear GarML without inteins (pCirc-GarML), and another for the production of the linear S1-F60 GarML flanked by fragments I_C_ and I_N_ with one amino acid substitution each, N36D and C1A, respectively (pCirc-Npu*mut*-GarML). These two point mutations are known to be sufficient for the loss of its protein-splicing function (Cheriyan et al., 2013).

### *In vitro* cell-free protein synthesis, evaluation of the antimicrobial activity of the pCirc-derived garvicin ML constructs, and determination of the molecular mass of the produced garvicin ML

**Figure 2:**
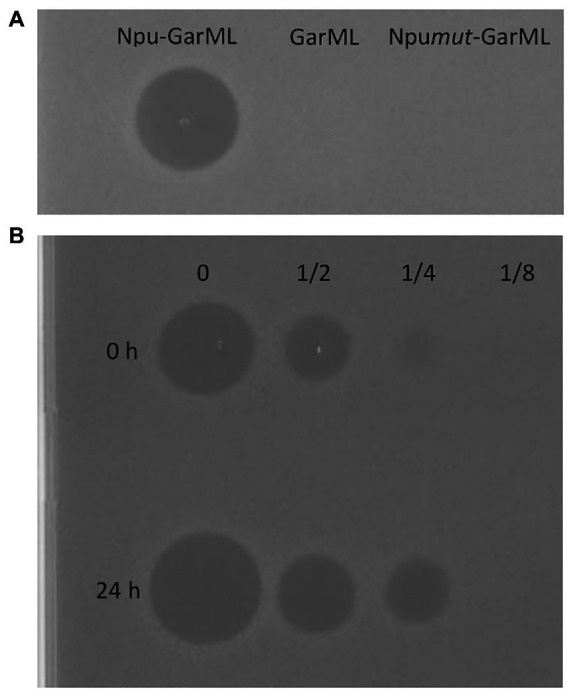
Antimicrobial activity of the IV-CFPS-produced-pCir-derived gene products against *L*. *garvieae* CECT5806. **(A)** Antimicrobial activity of the Npu-GarML and Npumut-SarML gene products. **(B)** Antimicrobial activity of the Npu-GarML gene product right after the *in vitro* CFPS-derived production (0 h) or after a 24 h incubation of the samples at room temperature.

**Figure 3:**
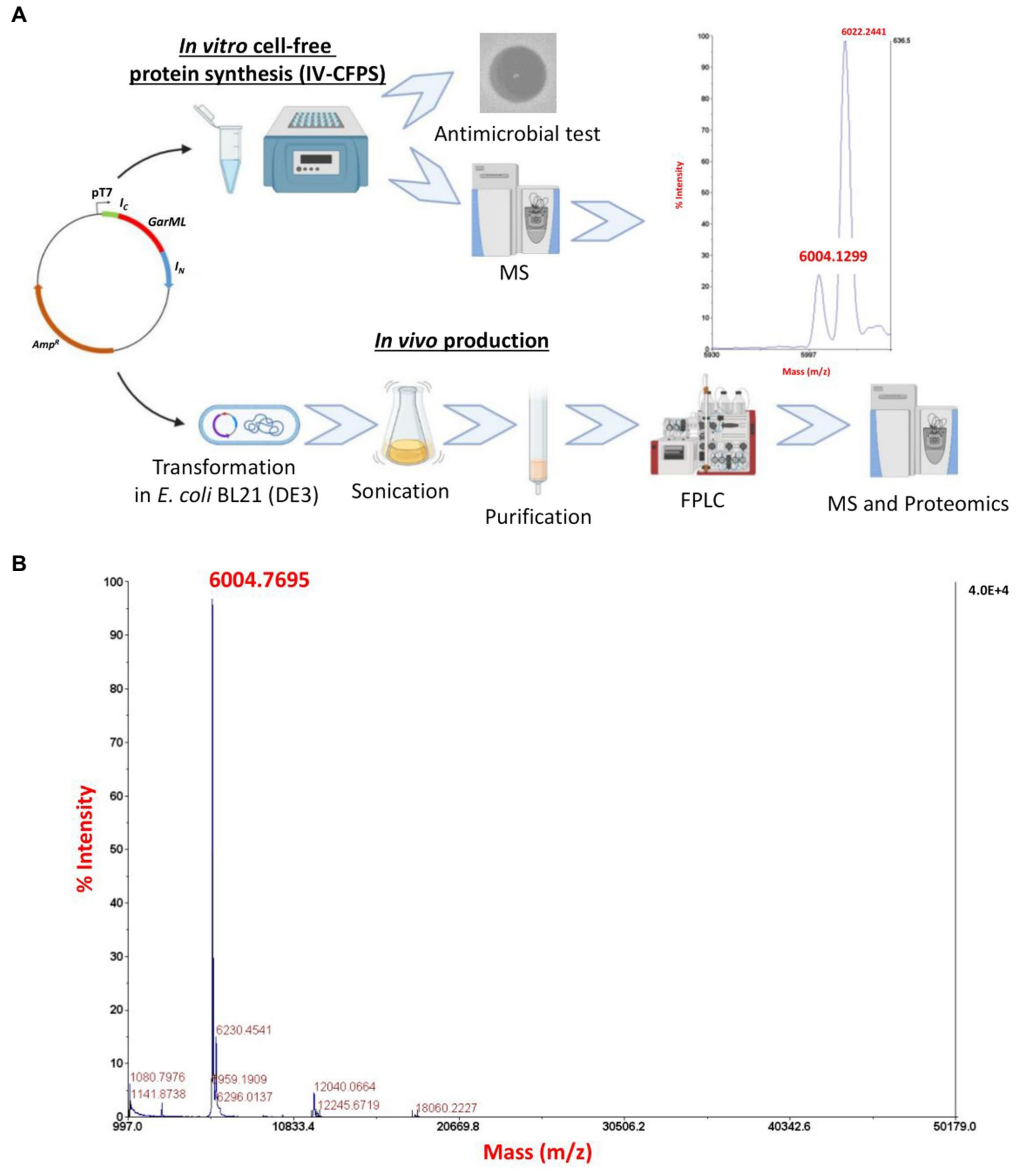
**(A)** Schematic representation of the *in vitro* and *in vivo* production and split-intein mediated ligation (SIML) of GarML, evaluation of the antimicrobial activity and MALDI-TOF MS analysis of the *in vitro* CFPS-produced GarML. **(B)** MALDI-TOF MS analysis of the in vivo produced GarML in the cellular soluble fraction (CFS) of *E*. *coli* BL21 (DE3), transformed with pCirc-Npu-GarML.

Plasmids pCirc-Npu-GarML, pCirc-GarML and pCirc-Npu*mut*-GarML were used as templates for the IV-CFPS of the Npu-GarML, GarML and Npu*mut*-GarML encoded peptides, respectively. The antimicrobial activity of the samples was tested against a GarML-sensitive indicator, *L. garvieae* CECT5806. Neither GarML nor Npu*mut*-GarML constructs showed a detectable antimicrobial activity (Figure 2A), suggesting that the designed linear versions of GarML, with mutated inteins or without inteins, do not possess antimicrobial activity. However, the *in vitro* CFPS-produced Npu-GarML peptides showed antagonistic activity against *L. garvieae* CECT5806 (Figure 2A), strongly suggesting that the SIML of GarML occurred. This antagonistic activity was higher when the *in vitro* reaction was left overnight before running the antimicrobial activity assay (Figure 2B). Confirmation of splicing of the inteins in the IV-CFPS and circularization of GarML, was further obtained by matrix-assisted laser desorption ionization–time of flight mass spectrometry (MALDI-TOF MS) analysis. The results showed the presence, in the evaluated sample, of a peptide with the exact molecular mass (6004.2) of the native GarML (Figure 3A), produced by *L. garvieae* DCC43, thus suggesting that SIML of residues S1 and F60 from GarML occurred as predicted, leaving no extra amino acid residues in the IV-CFPS produced mature GarML.

### *In vivo* production and characterization of the garvicin ML produced by recombinant *Escherichia coli* cells

Besides the IV-CFPS combined with the SIML of the linearly designed GarML, the *in vivo* production and circularization of the bacteriocin GarML was also determined from *E. coli* BL21 (DE3) (pCir-Npu-GarML), as the heterologous producer host. After growth and IPTG induction of the *E. coli* recombinant cells, the resulting cellular soluble fraction (CSF), derived from sonication of the grown cells, showed antimicrobial activity against *L. garvieae* CECT5806. The antimicrobial activity in the CSF was further purified by hydrophobic interaction and reversed-phase fast-performance liquid chromatography (RP-FPLC; Figure 3A). The antimicrobial activity of the fractions correlated with proteins or peptides eluting at 39% isopropanol, after two consecutive rounds of purification by RP-FPLC (results not shown). MALDI-TOF MS analysis of the purified active fraction showed major peptide fragments of a molecular mass (Figure 3B), that correlated with that of the native circular GarML (6004.2) produced by *L. garvieae* DCC43 (Borrero et al., 2011). The active purified fraction was further subjected to trypsin digestion and the resulting peptide fragments evaluated by targeted proteomics combined with massive peptide analysis. All precursor peptides from the GarML amino acid sequence were confirmed by MS/MS, covering 100% of the sequence. One of the fragments identified and confirmed by MS/MS, contained the amino acid residues S1-F60, from the Npu-GarML construct linked together (TIVNAVSAGMDIATALSFSGAFTAAGGIMALIKK) confirming the circularization of the purified GarML (Table 3).

**Table 3:** Peptide fragments, identified by targeted proteomics combined with massive peptide analysis of the purified GarML in the RP-FPLC-derived eluates from the cellular soluble fraction (CSF) of the *E. coli* (DE3) (pCir-Npu-GarML) cultures.

Peptide sequence	Modifications	Theoretical MH+ [Da]	Found in sample	Detected m/z
QLIAALVATG**M**AAGVAK	1 oxidation (M11)	1,600,91	High	800,95
QLIAALVATGMAAGVAK		1,584,91	High	792,96
IAALVATG**M**AAGVAK	1 oxidation (M11)	1,359,77	High	680,38
LVATG**M**AAGVAK	1 oxidation (M6)	1,104,61	High	552,80
LVATGMAAGVAK		1,088,61	Not found	
YAQKKLWK		1,064,63	High	532,81
TIVNAVSAG**M**DIATALSLFSGAFTAAGGIMALIKK	1 oxidation (M10)	3,426,84	High	857,46
TIVNAVSAGMDIATALSLFSGAFTAAGGIMALIK		3,282,75	High	1,094,92
TIVNAVSAG**M**DIATALSLFSGAFTAAGGIMALIK	1 oxidation (M10)	3,298,74	High	1,100,25
TIVNAVSAG**M**DIATALSLFSGAFTAAGGI**M**ALIK	2 oxidations (M10;M30)	3,314,74	High	1,105,58

Methionine residues are shown in bold when oxidized.

### *In vitro* cell-free protein synthesis, split-intein mediated ligation and antimicrobial activity of well characterized and still uncharacterized circular bacteriocins

During this work we have also proceeded to the design of gene constructs encoding the Npu-derived-I_C_ and I_N_ intein fragments, flanking the mature amino acid sequence of a set of well characterized and still uncharacterized putative circular bacteriocins (i.e., bacteriocin candidate genes identified by bioinformatic screening). The amino acid residues in the C-terminal and N-terminal joints, fused to the Npu-derived-I_C_ and I_N_ intein fragments, were selected from their native mature bacteriocins so they all would start with a serine (S) in position 1 (Supplementary material). In addition a protein degradation tag (SsrA) was included at the C-terminus of the gene constructs. As performed for production of GarML, a pCirc-derived bacteriocin expression vector without inteins was designed for all selected mature bacteriocins. All plasmids from the genetic constructs were used as templates for the IV-CFPS and SIML of the selected circular bacteriocins. The antimicrobial activity of the samples during overnight incubation at room temperature, was tested against the bacteriocin-sensitive indicator *L. lactis* IL1403. None of the control gene constructs, encoding known or putative mature circular bacteriocins without inteins showed antimicrobial activity when produced by the IV-CFPS and SIML procedure (results not shown), thus implying that proper intein-splicing and circularization are needed to produce an active antimicrobial peptide. From the pCirc-Npu-circular bacteriocin constructs, 10 out the 13 characterized bacteriocins showed a clear antimicrobial activity, while only 5 out of the 13 putative circular bacteriocins showed antimicrobial activity against *L. lactis* IL1403 (Table 4).

**Table 4:** Antimicrobial activity of the bacteriocins produced by the *in vitro* CFPS and split-intein mediated ligation (SIML) of circular bacteriocins against *L. lactis* IL1403. Fully characterized bacteriocins, in bold.

Bacteriocin	Producer	Plasmid	Activity against *L. lactis* IL1403*
			
Amylocyclicin	*Bacillus amyloliquefaciens* FZB42	pCirc-Npu-Alc	+
Enterocin AS-48	*Enterococcus faecalis* S-48	pCirc-Npu-EntAS48	+
Carnocyclin A	*Carnobacterium maltaromaticum* UAL307	pCirc-Npu-CarA	+
Circularin A	*Clostridium beijerinckii* ATCC 25752	pCirc-Npu-CirA	+
Enterocin NKR-5-3B	*Enterococcus faecium* NKR-5-3	pCirc-Npu-NKR_5_3B	+
Garvicin ML	*Lactococcus garvieae* DCC43	pCirc-Npu-GarML	+
Leucocyclicin Q	*Leuconostoc mesenteroides* TK41401	pCirc-Npu-LeuQ	+
Uberolysin A	*Streptococcus uberis* 42	pCirc-Npu-UberA	−
Butyrivibriocin AR10	*Butyrivibrio fibrisolvens* AR10	pCirc-Npu-ButAR10	−
Paracyclicin P	*Lactobacillus paracesei* JCM 8130/ DSM 5622	pCirc-Npu-ParP	+
Gassericin A	*Lactobacillus gasseri* LA39	pCirc-Npu-GasA	+
Plantaricyclin A	*Lactobacillus plantarum* NI326	pCirc-Npu-PlcA	+
Cerecyclin	*Bacillus* sp. Xin1	pCirc-Npu-Cer	−
Bacteriocin 3688STDY6124959	*Staphylococcus aureus* 3688STDY6124959	pCirc-Npu-3688STDY	+
Bacteriocin BCW 2997	*Listeria monocytogenes* BCW 2997	pCirc-Npu-BCW_2,997	+
Bacteriocin CF11	*Clavibacter michiganensis* CF11	pCirc-Npu-CF11	−
Bacteriocin NBRC 15376	*Paenibacillus chondroitinus* NBRC 15376	pCirc-Npu-NBRC_15,376	−
Bacteriocin YS111	*Streptococcus suis* YS111	pCirc-Npu-YS111	+
Bacteriocin DSM 15102	*Garciella nitratireducens* DSM 15102	pCirc-Npu-DSM_15,102	+
Bacteriocin AFS089278	*Bacillus toyonensis* AFS089278	pCirc-Npu-AFS089278	−
Bacteriocin TD3	*Bacillus vallismortis* TD3	pCirc-Npu-TD3	−
Bacteriocin NRRL B-24287	*Streptomyces pathocidini* NRRL B-24287	pCirc-Npu-NRLL_B_24,287	−
Bacteriocin AK22	*Alkalibacterium* AK22	pCirc-Npu-AK22	−
Bacteriocin 15,828	*Gemella cuniculi* DSM 15828	pCirc-Npu-15,828	−
Bacteriocin NCTC 12958	*Streptococcus thermophilus* NCTC 12958	pCirc-Npu-NCTC_12,958	+
Bacteriocin UoS2029	*Streptococcus pneumoniae* UoS2029	pCirc-Npu-UoS2029	−

* Samples showing a clear halo of inhibition (+) or no halo of inhibition (−).

## Discussion

In a previous study we described the use of IV-CFPS methodologies for the production of mature class II bacteriocins, including the construction of a standarized synthetic gene library (PARAGEN 1.0 collection) for the synthesis of both, fully characterized and putative not yet well-characterized class II bacteriocin candidates (Gabant and Borrero, 2019). The use of IV-CFPS-derived methodologies are ideal for testing the antimicrobial activity of putative new bacteriocins. Most importantly, these systems allow for the production of mature bacteriocins without the requirement of any other proteins, encoded in the bacteriocin gene clusters and involved in their processing, transport, regulation and immunity. However, although IV-CFPS-derived methods seems to be appropriate for production of the class II non-modified bacteriocins, these methods are not so efficient for production of the class I circular bacteriocins and other class I-derived bacteriocins from the group of RiPPs (Montalbán-López et al., 2021; Ongpipattanakul et al., 2022), requiring post-translational proccessing and modifications for their antimicrobial activity.

Circular bacteriocins are considered as one of the most promising groups of antimicrobial peptides for industrial applications, due to their higher stability compared to their linear counterparts (Gabrielsen et al., 2014; Perez et al., 2017). Until now, circular bacteriocins remained as a quite selective group of the class I bacteriocins with about 21 antimicrobial peptides identified and fully characterized. However, due to recent advances in the *omic* technologies, especially in genome sequencing, a high number of putative circular bacteriocin clusters have been identified in the genomes of Gram positive bacteria posted in different databases, suggesting these peptides are more prevalent in nature than previously expected (Vezina et al., 2020; Xin et al., 2020; Major et al., 2021). However, in the absence of the producer strains it is difficult to evaluate the synthesis, production and, most importantly, the antimicrobial activity of these bacteriocins against a number of microbial indicators.

In this work we have determined the potential of the IV-CFPS system combined with the split-intein mediated circularization of peptides and proteins (SICLOPPS), for evaluation of the synthesis, production and antimicrobial activity of circular bacteriocins. The use of inteins for peptide circularization was first described by Scott et al. (1999), and since then, a number of peptides and proteins have been circularized and tested for their biological activities. During the last few years, there has been also an important effort for improving this technology through the evaluation of more efficient and faster inteins, the incorporation of tags for rapid chromatographic column purification of the circularized peptides, and strategies for reduction of peptide and protein degradation. Accordingly, we have used this methodology by using an improved version that incorporates an Npu DnaE split intein from the filamentous cyanobacteria *Nostoc punctiforme,* which is faster and more tolerant to amino acid variations in peptide sequences, specially near the splice junctions, as well as a protein degradation tag (SsrA sequence) in the C-terminus of the designed gene constructions to target the spliced Npu inteins to the ClpXP complex, an ATP-powered unfolding and protein-degradation machine for degradation and elimination of their potential *in vivo* toxicity (Townend and Tavassoli, 2016).

The IV-CFPS and split-intein mediated ligation (SIML) of circular bacteriocins was first evaluated with the bacteriocin GarML, a well characterized circular bacteriocin produced by *L. garvieae* DCC43, isolated from mallard ducks (*Anas platyrhynchos*; Borrero et al., 2011). GarML is synthesized as a 63-amino-acid precursor peptide which is processed to produce the 60-amino-acid mature peptide. The putative leader peptide (tripeptide) of the GarML precursor is cleaved off and cyclization takes place between the N-terminal leucine (L4) and the C-terminal alanine (A60) by a peptide bond. The predicted secondary structure of GarML suggests that GarML folds into a compact globular bundle comprised of four conserved α-helices enclosing a compact hydrophobic core (Borrero et al., 2011). The maltose ABC transporter potentially functions as a target receptor of GarML, rendering the permease open for the efflux of intracellular solutes, eventually leading to cell death (Gabrielsen et al., 2012). However, understanding of the cyclization processes for GarML and other circular bacteriocins is still poorly understood, although it is suggested that leader peptide removal and cyclization are two separate processes (Gabrielsen et al., 2014; Perez et al., 2017).

Since splicing by the Npu intein is more efficient when a cysteine (C) or a serine (S) residues is in position +1 of the linear peptide, the selection of the S32 residue as the +1 residue in the designed novel GarML gene construct over the other two serine residues present in mature GarML (S19 and S29), was arbitrary (Townend and Tavassoli, 2016). Thus, a synthetic gene containing the C-and N-terminal intein fragments of the Npu split intein (I_C_ and I_N_, respectively), flanking the sequence of the novel mature GarML was synthesized. In addition, a protein degradation tag (SsrA) was included in the C-terminus of the gene construct to overcome the potential toxicity of the Npu intein after splicing. Once the complete amino acid sequence the novel GarML was designed (S1-F60) according to the codon usage of *E. coli*, it was placed under the control of a T7 promoter in a pUC-derived protein expression vector (pCirc-Npu-GarML). In addition, two control pUC-derived constructs were synthesized: one for the synthesis of the S1-F60 GarML without inteins (pCirc-GarML), and another for the production of S1-F60 GarML flanked by the I_C_-and I_N_-derived inteins with one amino acid substitution each, N36D and C1A, respectively (pCirc-Npu*mut*-GarML). These two point mutations are known to be sufficient for the loss of the protein-splicing function (Cheriyan et al., 2013; Figure 1B).

The plasmid pCirc-constructs were then used as templates for the IV-CFPS of the encoded peptides. When the antimicrobial activity of the synthesized peptides were tested against a GarML-sensitive indicator neither samples, derived from the pCirc-GarML or the pCirc-Npu*mut*-GarML gene constructs, showed a detectable antagonistic activity (Figure 2A), suggesting that linear versions of the GarML do not possess antimicrobial activity. However, the pCirc-Npu-GarML template allowed the synthesis of a peptide that showed a potent antimicrobial activity against the *L. garvieae* CECT5806 indicator (Figure 2A), thus strongly suggesting that SIML of GarML occurred. Traceless SIML of GarML was further confirmed by MALDI-TOF MS (Figure 3A). Interestingly, a higher antimicrobial activity was observed when the IV-CFPS-derived production of GarML was left overnight at room temperature. Whereas the split-intein mediated circularization of peptides and proteins has been estimated to occur within the first 30–60 s of the reaction (Townend and Tavassoli, 2016), it could be suggested that the observed higher antimicrobial activity of the samples would be attributed to a higher production of GarML, during its extended *in vitro* CFPS-derived production (Figure 2B).

However, although the IV-CFPS-production and SIML of the bacteriocin GarML, could be considered as an efficient and fast method for its production and testing, the high cost per μl reaction is one of the main drawbacks of this technology, especially for those situations where a higher production of the protein is needed. Accordingly, the pCirc-Npu-GarML construct was further evaluated for the *in vivo* production of GarML by *E. coli*, as the heterologous producer host. After growth and induction of the recombinant *E. coli* BL21 (DE3) (pCirc-GarML) host, the antimicrobial activity of its cellular soluble fraction (CSF) revealed the presence of peptide fragments of a molecular mass that correlated with that of the native circular GarML (Figure 3B). The trypsin digestion and targeted proteomics combined with massive peptide analysis of the resulting peptide fragments revealed the presence, in the purified samples, of 6 peptides covering 100% of the mature amino acid sequence of GarML. Furthermore, one of the resulting fragments (TIVNAVSAGMDIATALSFSGAFTAAGGIMALIKK) included the amino acid residues S1 and F60 linked together, thus confirming the split-intein mediated ligation (SIML) and head-to-tail circularization of the *in vivo* produced GarML (Table 3).

Interestingly enough the results obtained in this work suggest that modification of the circularization site, during the *in vitro* and *in vivo* production and SIML of mature GarML, permitted determination of the antimicrobial activity of the produced bacteriocin suggesting that, after ligation, GarML is most likely able to adopt an active tridimensional (3D) conformation spontaneously. This was also observed during the evaluation of the PARAGEN synthetic gene library for the IV-CFPS-mediated production of bacteriocins, where different class IIa pediocin-like bacteriocins, having 2 or 3 disulfide bonds in their native conformation, showed a similar antimicrobial activity and antagonistic spectrum against sensitive bacteria, than the produced by their native hosts (Gabant and Borrero, 2019).

As far as we know this is the first study showing that a circular bacteriocin such as the GarML is produced *in vivo* in the absence of additional and dedicated genes involved in its immunity, processing and secretion. However the heterologous production and recovery of GarML from *E. coli* is still far to be optimal. We have observed that cultures of *E. coli* BL21 (DE3), transformed with pCirc-Npu-GarML, do not grow at the same speed and do not reach the same OD_600_ after induction with IPTG, as the uninduced cultures (results not shown). This is probably due to a toxic effect derived from the accumulation of GarML inside the producer. Accordingly, the production and recovery of GarML in the cellular soluble fraction (CSF) of the *E. coli* producer most surely would be enhanced by the use of additional heterologous production hosts, by further improvement of the gene constructs designed for their production such as the addition of specific immunity genes, by the use of switchable inteins for conditional protein splicing or by using more efficient fusion tags to facilitate purification of the *in vivo* produced bacteriocins.

In this work, both the IV-CFPS and the SIML protocols for circularization of the bacteriocin GarML, have been also evaluated to direct the synthesis and production of 26 circular bacteriocins and for determination of their antimicrobial activity. 13 out of the 26 bacteriocins evaluated were well described circular bacteriocins including enterocin AS-48 and gassericin A, two well documented bacteriocins successfully tested in a broad range of biotechnological applications (Hu et al., 2018; Montalbán-López et al., 2020). The other 13 peptides selected for this study were putative circular bacteriocins recently identified from genome mining studies, but whose antimicrobial activity has not yet been experimentally confirmed (Vezina et al., 2020; Xin et al., 2020; Major et al., 2021). All the pUC-derived vectors, encoding the pCir-Npu-gene products for mature circular bacteriocins were used as templates for their IV-CFPS and SIML of the bacteriocins, and further assayed for their antimicrobial activity against *L. lactis* IL1403 as the potential sensitive bacterial indicator. As shown in Tables 4, 15 out of the 26 circular bacteriocins evaluated showed antimicrobial activity, including 5 bacteriocins of the group of the not yet experimentally confirmed putative circular bacteriocins. However, 8 of the samples evaluated did not show antimicrobial activity. We also cannot discard that the lack of antimicrobial activity, observed in some of the IV-CFS produced and SIML-derived circular bacteriocins, could be due to the predetermined selection of a microbial indicator (*L. lactis* IL1403), immune or insensitive to these bacteriocins. There are also no compelling reasons why these bacteriocins should be active against *Lactococcus* species not genetically related to the producer strains. Further experiments should be performed to confirm that the lack of antimicrobial activity of these samples may be due to a much lower *in vitro* peptide production, lack of SIML of the fused peptides and/or loss of an appropriate three-dimensional (3D) structure of the synthesized mature bacteriocins for recognition of specific or non-specific receptors for attachment to the *L. lactis* IL1403 indicator strain. It would be also interesting to determine whether the initial circularization site (serine selected) has an impact on the overall circularization, structure and activity of the IV-CFPS and SIML produced bacteriocins. We have also confirmed the antimicrobial activity of 5 out of 13 putative circular bacteriocins, whereas in the near future it will be interesting to determine their antimicrobial activity and spectrum of activity against a larger number of microorganisms of interest in the food industry, human and animal health.

## Conclusion

Here we describe an efficient synthetic biology tool for the *in vitro* and *in vivo* production and split-intein mediated ligation (SIML) of experimentally proven or putative circular bacteriocins, without the requirement of the native producer strain or the need of additional genes required for their immunity, regulation, processing and secretion. This work opens the door to further studies where inteins can be used for the *in vitro* and *in vivo* production, ligation, circularization and functional analysis not only of class I circular bacteriocins, but also of class II-derived bacteriocins with the aim of generating circularized peptides with, possibly, higher antimicrobial activity, stability and proteinase resistance. The described protocol may be also useful for generating bacteriocin libraries with novel bacteriocin-derived circular variants with enhanced biotechnological features.

## Data availability statement

The original contributions presented in the study are included in the article/supplementary material, further inquiries can be directed to the corresponding author.

## Author contributions

NP, MJB, ES, EMA, and IL were involved in experimental design, conducted experiments, and collected data. MEB designed the plasmids and gene synthesis. LMC contributed to experimental design. PEH contributed to experimental design and manuscript writing. PG and JB contributed to experimental design, project leadership, and manuscript writing. All authors contributed to the article and approved the submitted version.

## Funding

Financial support was provided by the Atracción de Talento Program of the Comunidad de Madrid [2018-T1/BIO-10158], Ministerio de Ciencia, Innovación y Universidades [PID2019-104808RA-I00] and article 83 (L.O.U.) contract between Syngulon SA and the UCM. NP, IL, and JB were supported by the Atracción de Talento Program of the Comunidad de Madrid [2018-T1/BIO-10158]. MJB was supported by the EVOL-MODEL grant n° 7893 from the Region Wallonne, and ES was supported by the Empleo Juvenil Program of the Comunidad de Madrid [PEJ-2020-AI/BIO-17758].

## Conflict of interest

MJB, MEB, and PG were employed by company Syngulon SA.

The remaining authors declare that the research was conducted in the absence of any commercial or financial relationships that could be construed as a potential conflict of interest.

## Publisher’s note

All claims expressed in this article are solely those of the authors and do not necessarily represent those of their affiliated organizations, or those of the publisher, the editors and the reviewers. Any product that may be evaluated in this article, or claim that may be made by its manufacturer, is not guaranteed or endorsed by the publisher.
