# Supplementary material for: In vitro and in vivo production and split-intein mediated ligation (SIML) of circular bacteriocins
Source: Front Microbiol. 2022 Nov 14;13:1052686. doi: 10.3389/fmicb.2022.1052686 (PMC9703936; doi:10.3389/fmicb.2022.1052686)
Supplement: Supplementary file 1 [file Table_1.docx]

Table 1. Bacteriocins evaluated for the *in vitro* CFPS-production and split-intein mediated ligation (SIML) of circular bacteriocins. Bacteriocins fully characterized, in bold. The first line in the mature amino acid sequence of the bacteriocins corresponds to their experimentally determined or putative linear sequence, after leader sequence cleavage and before head-to-tail circularization. The second line, shadowed in grey, corresponds to the linear amino sequence used in this study for their split-intein mediated ligation (SIML) and circularization. The serine designed for position +1 in the pUC-derived gene constructs, in red.

| **Bacteriocin** | **Producer strain** | **Mature amino acid sequence** | **Reference** |
| --- | --- | --- | --- |
| **Amylocyclicin** | *Bacillus amyloliquefaciens* FZB42 | LASTLGI**S**TAAAKKAIDIIDAASTIASIISLIGIVTGAGAISYAIVATAKTMIKKYGKKYAAAW STAAAKKAIDIIDAASTIASIISLIGIVTGAGAISYAIVATAKTMIKKYGKKYAAAWLASTLGI | (1) |
| Bacteriocin 3688STDY6124959 | *Staphylococcus aureus* 3688STDY6124959 | LT**S**TLGISSYAAKKAIDIIAAAGDVAAIVGLIGAVTGAGAIGAGILFTAKKLIKSYGKKYAAAW STLGISSYAAKKAIDIIAAAGDVAAIVGLIGAVTGAGAIGAGILFTAKKLIKSYGKKYAAAWLT | (2) |
| **Enterocin AS-48** | *Enterococcus faecalis* S-48 | MAKEFGIPAAVAGTVLNVVEAGGWVTTIVSILTAVGSGGLSLLAAAGRE**S**IKAYLKKEIKKKGKRAVIAW  SIKAYLKKEIKKKGKRAVIAWMAKEFGIPAAVAGTVLNVVEAGGWVTTIVSILTAVGSGGLSLLAAAGRE | (3) |
| Bacteriocin BCW 2997 | *Listeria monocytogenes* BCW 2997 | LAKEFGIPGGVAATVLNIVEAGGWATTILSILTAVGSGGLSLIAAAGKE**S**IKVYLKKQIKKKGRKAVIAW  SIKVYLKKQIKKKGRKAVIAWLAKEFGIPGGVAATVLNIVEAGGWATTILSILTAVGSGGLSLIAAAGKE | (2) |
| Bacteriocin CF11 | *Clavibacter michiganensis* CF11 | LAQQFGIP**S**AVGGTILNLATAGGTAATIVGILVGLGSGGLGLLAAAGRETIKQFLLNEIKKKGRKAVIAW  SAVGGTILNLATAGGTAATIVGILVGLGSGGLGLLAAAGRETIKQFLLNEIKKKGRKAVIAWLAQQFGIP | (2) |
| Bacteriocin NBRC 15376 | *Paenibacillus chondroitinus* NBRC 15376 | LAKEFGVPAVVGNALVQALDWGLTAATIA**S**IIGAFITGGLSIVAAAGTTALKTYLRNKLAEMGTKAFVAW  SIIGAFITGGLSIVAAAGTTALKTYLRNKLAEMGTKAFVAWLAKEFGVPAVVGNALVQALDWGLTAATIA | (2) |
| **Carnocyclin A** | *Carnobacterium maltaromaticum* UAL307 | LVAYGIAQGTAEKVV**S**LINAGLTVGSIISILGGVTVGLSGVFTAVKAAIAKQGIKKAIQL  SLINAGLTVGSIISILGGVTVGLSGVFTAVKAAIAKQGIKKAIQLLVAYGIAQGTAEKVV | (4) |
| **Circularin A** | *Clostridium beijerinckii* ATCC 25752 | VAGALGVQTAAATTIVNVILNAGTLVTVLGIIA**S**IASGGAGTLMTIGWATFKATVQKLAKQSMARAIAY  SIASGGAGTLMTIGWATFKATVQKLAKQSMARAIAYVAGALGVQTAAATTIVNVILNAGTLVTVLGIIA | (5) |
| Bacteriocin YS111 | *Streptococcus suis* YS111 | VAAALGVPSAVA**S**TVVNVILGAGTAVTILGIISSIASGGATTLLTVGWAGFKATVQRLAKQSMARAIAY  STVVNVILGAGTAVTILGIISSIASGGATTLLTVGWAGFKATVQRLAKQSMARAIAYVAAALGVPSAVA | (2) |
| **Enterocin NKR-5-3B** | *Enterococcus faecium* NKR-5-3 | LTANLGI**S**SYAAKKVIDIINTGSAVATIIALVTAVVGGGLITAGIVATAKSLIKKYGAKYAAAW  SSYAAKKVIDIINTGSAVATIIALVTAVVGGGLITAGIVATAKSLIKKYGAKYAAAWLTANLGI | (6) |
| Bacteriocin DSM 15102 | *Garciella nitratireducens* DSM 15102 | LA**S**MLGISTVAAKKVIDIIDTASTVATIISLIGVIVGVGGITAGLVTTAKAMIKKYGKKYATMW  SMLGISTVAAKKVIDIIDTASTVATIISLIGVIVGVGGITAGLVTTAKAMIKKYGKKYATMWLA | (2) |
| **Garvicin ML** | *Lactococcus garvieae* DCC43 | LVATGMAAGVAKTIVNAVSAGMDIATALSLF**S**GAFTAAGGIMALIKKYAQKKLWKQLIAA  SGAFTAAGGIMALIKKYAQKKLWKQLIAALVATGMAAGVAKTIVNAVSAGMDIATALSLF | (7) |
| **Leucocyclicin Q** | *Leuconostoc mesenteroides* TK41401 | LVNQLGI**S**KSLANTILGAIAVGNLASWLLALVPGPGWATKAALATAETIVKHEGKAAAIAW  SKSLANTILGAIAVGNLASWLLALVPGPGWATKAALATAETIVKHEGKAAAIAWLVNQLGI | (8) |
| Bacteriocin AFS089278 | *Bacillus toyonensis* AFS089278 | LINKLGI**S**KSLAQDILTAIAVGNVASWLLVLIPGPGWATKAAISAAEIIVETSGEAAAVAY  SKSLAQDILTAIAVGNVASWLLVLIPGPGWATKAAISAAEIIVETSGEAAAVAYLINKLGI | (2) |
| **Uberolysin A** | *Streptococcus uberis* 42 | LAGYTGIA**S**GTAKKVVDAIDKGAAAFVIISIISTVISAGALGAVSASADFIILTVKNYISRNLKAQAVIW  SGTAKKVVDAIDKGAAAFVIISIISTVISAGALGAVSASADFIILTVKNYISRNLKAQAVIWLAGYTGIA | (9) |
| Bacteriocin TD3 | *Bacillus vallismortis* TD3 | VAGVL**S**VDKGVATKVVNAVDAAGWAFVGGSTLLAIISGGTLSAASISIDALIYTIKNYLKRSLKAQAIAW  SVDKGVATKVVNAVDAAGWAFVGGSTLLAIISGGTLSAASISIDALIYTIKNYLKRSLKAQAIAWVAGVL | (2) |
| Bacteriocin NRRL B-24287 | *Streptomyces pathocidini* NRRL B-24287 | ATTPNVLTGYFGLTGATANRVLNAIEAGTDIAAAL**S**VLGGVSAAGGVAMWMLKQAIAKGGRKAVVA  SVLGGVSAAGGVAMWMLKQAIAKGGRKAVVAATTPNVLTGYFGLTGATANRVLNAIEAGTDIAAAL | (2) |
| Bacteriocin AK22 | *Alkalibacterium* AK22 | ILFVADLFGIPFTTDIVDQVWGYIEGGAGTVTAI**S**WVLGVTIPAWAVPIIGAVGVVSA  SWVLGVTIPAWAVPIIGAVGVVSAILFVADLFGIPFTTDIVDQVWGYIEGGAGTVTAI | (2) |
| Bacteriocin 15828 | *Gemella cuniculi* DSM 15828 | VYFIAGKLGITLAPGWYEKLVNYI**S**AGGGAVDGVAMILGITLPGWAITVIGAFGLVSA  SAGGGAVDGVAMILGITLPGWAITVIGAFGLVSAVYFIAGKLGITLAPGWYEKLVNYI | (2) |
| **Butyrivibriocin AR10** | *Butyrivibrio fibrisolvens* AR10 | IYFIADKMGIQLAPAWYQDIVNWV**S**AGGTLTTGFAIIVGVTVPAWIAEAAAAFGIASA  SAGGTLTTGFAIIVGVTVPAWIAEAAAAFGIASAIYFIADKMGIQLAPAWYQDIVNWV | (10) |
| **Paracyclicin P** | *Lactobacillus. paracesei* subsp. *paracasei* JCM 8130/ DSM 5622 | IYFIANKLGIHLAPGWYQDMVNYV**S**AGGSLAGAFSVVAGVTLPAWIVPIATAFGAVSA  SAGGSLAGAFSVVAGVTLPAWIVPIATAFGAVSAIYFIANKLGIHLAPGWYQDMVNYV | (11) |
| Bacteriocin NCTC 12958 | *Streptococcus thermophilus* NCTC 12958 | IYWICGKFGIRLAPGWYQDIVDFV**S**AGGSIVDAFAIIAGITLPAWVAPVLAGFGVVSA  SAGGSIVDAFAIIAGITLPAWVAPVLAGFGVVSAIYWICGKFGIRLAPGWYQDIVDFV | (2) |
| Bacteriocin UoS2029 | *Streptococcus pneumoniae* UoS2029 | IYWVCGKFGIELAPGWYQDIVDFV**S**SGGTIVEAFAAVAGVTLPAWVGPVLAAFGLASA  SSGGTIVEAFAAVAGVTLPAWVGPVLAAFGLASAIYWVCGKFGIELAPGWYQDIVDFV | (2) |
| **Gassericin A** | *Lactobacillus gasseri* LA39 | IYWIADQFGIHLATGTARKLLDAMA**S**GASLGTAFAAILGVTLPAWALAAAGALGATAA  SGASLGTAFAAILGVTLPAWALAAAGALGATAAIYWIADQFGIHLATGTARKLLDAMA | (12) |
| **Plantaricyclin A** | *Lactobacillus plantarum* NI326 | IVWIAKQFGVHLTT**S**LTQKALDLLSAGSSLGTVAAAVLGVTLPAWAVAAAGALGGTAA  SLTQKALDLLSAGSSLGTVAAAVLGVTLPAWAVAAAGALGGTAAIVWIAKQFGVHLTT | (13) |
| **Cerecyclin** | *Bacillus* sp. Xin1 | VV**S**KLGWTGINIGTANALIGALMTGSDIWTAISVAGLAFGGGIGTAISTIGRKAIMEMVEKVGKKKAAQW  SKLGWTGINIGTANALIGALMTGSDIWTAISVAGLAFGGGIGTAISTIGRKAIMEMVEKVGKKKAAQWVV | (14) |

**REFERENCES**

1. Scholz R, Vater J, Budiharjo A, Wang Z, He Y, Dietel K, Schwecke T, Herfort S, Lasch P, Borriss R. Amylocyclicin, a novel circular bacteriocin produced by *Bacillus amyloliquefaciens* FZB42. J Bacteriol (2014) 196 (10):1842–52. doi: 10.1128/JB.01474-14

2. Vezina B, Rehm BHA, Smith AT. Bioinformatic prospecting and phylogenetic analysis reveals 94 undescribed circular bacteriocins and key motifs. BMC Microbiol (2020) 20 (1):77. doi: 10.1186/s12866-020-01772-0

3. Gálvez A, Maqueda M, Martínez-Bueno M, Valdivia E. Bactericidal and bacteriolytic action of peptide antibiotic AS-48 against gram-positive and gram-negative bacteria and other organisms. Res Microbiol (1989) 140 (1):57–68. doi: 10.1016/0923-2508(89)90060-0

4. Martin-Visscher LA, van Belkum MJ, Garneau-Tsodikova S, Whittal RM, Zheng J, McMullen LM, Vederas JC. Isolation and characterization of carnocyclin a, a novel circular bacteriocin produced by *Carnobacterium maltaromaticum* UAL307. Appl Environ Microbiol (2008) 74 (15):4756–63. doi: 10.1128/AEM.00817-08

5. Kemperman R, Kuipers A, Karsens H, Nauta A, Kuipers O, Kok J. Identification and characterization of two novel clostridial bacteriocins, circularin A and closticin 574. Appl Environ Microbiol (2003) 69 (3):1589–97. doi: 10.1128/AEM.69.3.1589-1597.2003

6. Himeno K, Rosengren KJ, Inoue T, Perez RH, Colgrave ML, Lee HS, et al. Identification, characterization, and three-dimensional structure of the novel circular bacteriocin, enterocin nkr-5-3b, from *Enterococcus faecium*. Biochemistry (2015) 54 (31):4863–76. doi: 10.1021/acs.biochem.5b00196

7. Borrero J, Brede DA, Skaugen M, Diep DB, Herranz C, Nes IF, Cintas LM, Hernández PE. Characterization of garvicin ML, a novel circular bacteriocin produced by *Lactococcus garvieae* DCC43, isolated from mallard ducks (Anas platyrhynchos). Appl Environ Microbiol (2011) 77 (1):369–73. doi: 10.1128/AEM.01173-10

8. Masuda Y, Ono H, Kitagawa H, Ito H, Mu F, Sawa N, Zendo T, Sonomoto K. Identification and characterization of leucocyclicin Q, a novel cyclic bacteriocin produced by *Leuconostoc mesenteroides* TK41401. Appl Environ Microbiol (2011) 77 (22):8164–70. doi: 10.1128/AEM.06348-11

9. Wirawan RE, Swanson KM, Kleffmann T, Jack RW, Tagg JR. Uberolysin: a novel cyclic bacteriocin produced by *Streptococcus uberis*. Microbiology (2007) 153 (Pt 5):1619–30. doi: 10.1099/mic.0.2006/005967-0

10. Kalmokoff ML, Cyr TD, Hefford MA, Whitford MF, Teather RM. Butyrivibriocin AR10, a new cyclic bacteriocin produced by the ruminal anaerobe *Butyrivibrio fibrisolvens* AR10: characterization of the gene and peptide. Can J Microbiol (2003) 49 (12):763–73. doi: 10.1139/w03-101

11. Collins FWJ, O’Connor PM, O’Sullivan O, Gómez-Sala B, Rea MC, Hill C, Ross RP. Bacteriocin Gene-Trait matching across the complete Lactobacillus Pan-genome. Sci Rep (2017) 7 (1):3481. doi: 10.1038/s41598-017-03339-y

12. Kawai Y, Saito T, Toba T, Samant SK, Itoh T. Isolation and characterization of a highly hydrophobic new bacteriocin (gassericin A) from *Lactobacillus gasseri* LA39. Biosci Biotechnol Biochem (1994) 58 (7):1218–21. doi: 10.1271/bbb.58.1218

13. Borrero J, Kelly E, O’Connor PM, Kelleher P, Scully C, Cotter PD, Mahony J, van Sinderen D. Plantaricyclin A, a novel circular bacteriocin produced by *Lactobacillus plantarum* NI326: purification, characterization, and heterologous production. Appl Environ Microbiol (2018) 84 (1). doi: 10.1128/AEM.01801-17

14. Xin B, Liu H, Zheng J, Xie C, Gao Y, Dai D, Peng D, Ruan L, Chen H, Sun M. In silico analysis highlights the diversity and novelty of circular bacteriocins in sequenced microbial genomes. mSystems (2020) 5 (3). doi: 10.1128/mSystems.00047-20
